# Book Notices and Reviews

**Published:** 1864-05

**Authors:** 


### BOOK NOTICES AND REVIEWS.

*Transactions of the American Medical Association. Vol.* 14, 1863.—The volume before us is about one-half the size of those that have preserved the transactions of former meetings of the Association. It contains the well-conceived and happily-written address of the acting President, Dr. Wilson Jewell, devoted to the consideration of the important question of public hygiene. In the report on Medical Education we are told that "the advancement in Medical Science and the increased value of its practical results are no longer debatable problems. To the sublime art of Medicine, as at present understood and practiced, humanity owes much of the physical welfare it now enjoys; as well as the diminished severity of disease and its control by intelligent and judicious treatment." In support of this it quotes as follows from Macaulay: "Every bricklayer who falls from a scaffold, every sweeper of a crossing who is run over by a carriage, now may have his wounds dressed and his limbs set with a skill, such as one hundred and sixty years ago, all the wealth of a great lord, like Ormond, or a merchant prince, like Clayton, could not have purchased. Some frightful diseases have been exterminated by science, and some have been banished by police. The term of human life has been lengthened over the whole kingdom,and especially in the towns. The year 1685 was not accounted sickly, yet in the year 1685 more than one in three of the inhabitants of the capital died. At present only one inhabitant of the capital in forty dies annually. The difference in salubrity between the London of the 19th century and the London of the 17th century is very far greater than the difference between London in an ordinary season and London in the cholera."

Yet the report goes on to say that "the profession nowhere has answered the full expectation of its early promise. It does not hold the proud position, especially in this country, to which, as the guardian of truth, it is really entitled." This deplorable condition of affairs is attributed, in the report, to two causes. First, to the "private practitioner, who enters the pupil upon the office register without inquiring of, or caring for his educational fitness for a calling which demands the highest order of intellect, and the most various study and acquirement. He is often destitute of the moral independence or conscience, or both, to reject an applicant he has reason to regard as unfit for the pursuit he has espoused." Second, "the professors of colleges, who graduate, annually, large classes of young men in the cities, who, it is not too much to affirm, are, for the most part, poorly furnished for the responsible duties awaiting them in the future. Each roll of parchment indorses its possessor as *vir ornatissimus*, and he goes forth an accredited agent—for life or death, endowed with all the paraphernalia of a Doctor of Medicine, but destitute of the brains." One would think this committee had forgotten that the multitude of worthy medical men who have crowned the profession with honor in times past, or who bless the present generation by their intelligent and humane labors, have found their way into the profession through the office instruction of these same "private practitioners," who are so severely censured, and have been publicly taught by "professors in colleges," who we are told, "annually graduate large classes of young men, destitute of brains." However, we may expect no more of this in future, for the committee have suggested the remedy: "Every physician who shall admit a young man into his office for instruction, without a certificate of suitable preliminary education," is to do so at the peril of being deemed "unprofessional, and liable of censure by, or expulsion from, any State or County Society to which he may belong." The entire system of college instruction is to be reformed. The number of professors is to be increased, and their appointment is to come from a Board of Regents, "a certain portion of whom should be medical men," the faculty of the college having no precedence in the nomination of candidates, or any influence, other than outsiders, in their selection. The custom of choosing the professors without regard to the wishes of the Faculty, and paying them from the public treasury, adopted in Europe, to insure as far as may be, a servile dependence of these institutions on the Government, from which they receive their support, is spoken of in terms that imply approbation.

There are to be not less than three courses of lectures of six months each, "and beyond this, a corps of teachers should be organized in connection with the Faculty to direct the studies of pupils during the recess of lectures." As every one knows the impossibility of keeping a class of medical students together after the close of the regular term of instruction, we are left to suppose that the duties of this "organization" are to be of an itinerary nature. The system of medical instruction pursued in Europe, appears to be the beau ideal of the committee, and we are informed that in "Edinburgh five years is the term of study, six months of each year, being passed in the University. In Paris, the number of professors is twenty-eight, besides assistant and adjunct professors, the course of instruction being embraced in eighteen courses of lectures, two a year. In Berlin, the number of professors and assistant professors is sixty-seven." Yet the example of Europe does not seem to us to offer much encouragement for the adoption of the views recommended in the report, for notwithstanding the great difficulty of obtaining a place in the profession there, we have no reason to suppose that the profession in Europe is more intelligent than it is in America, or that they treat disease more skillfully there than here.

The report pays a very high, and, as we think, merited compliment to Surgeon-General Hammond, to whom it says, "is due the credit of having inaugurated many wholesome reforms and stimulated a spirit of improvement unexcelled in its former history."

We think the report, taken as a whole, does the profession great injustice, and to speak of it as disparagingly as the report does, is as ungenerous as it is unjust, and such expression deliberately made and embodied in an authoritative announcement, which is adopted by a congress of medical men, might be expected to exert no small influence in robbing the profession of the "proud position to which it is entitled." The profession is not held, and does not deserve to be held, in light esteem in this country. It is as much honored and exerts as much and as beneficent an influence on the best interests of society as either of the kindred professions of Law, or Theology. A profession that has virtually exterminated small-pox, that has given us anæsthetics, that has enabled us to abridge the course, palliate the pain, greatly to curtail the mortality, or even by hygienic precautions, often entirely to escape from the great majority of diseases, that has, in a notable degree, lessened the perils of labor, and perfected the treatment of injuries, and in short been more potent than any single cause, in "lengthening the term of human life," should not be spoken of by any intelligent man, but least of all, by any member of the medical profession, in terms of disrespect. None will deny that there are incompetent and bad men in the profession. To do so would be to claim for it an exemption to which no profession nor class of society can pretend. But while this is true, it is equally true that the medical profession was never before more worthy of honor, and has never been as much enlightened, or as useful in its labors as it is to-day.

It has fully kept pace with the march of society, and has lent to the general advancement of the best interests of the race, a power not surpassed by any equal number of men in any vocation. The prime necessity for the advancement of society is found in the increase of its numbers, and none have exerted so great an influence in this respect as the medical profession, which has so greatly reduced the annual mortality of the race. The profession will undoubtedly continue to improve, in common with other branches of society, as man, by increased knowledge and virtue, shall acquire thereby increased control over the forces of nature. But this increase will not be the result of violent and radical changes. There was never yet a successful revolution that was not the fruit of a slow growth, and those who expect to reach perfection through violence, are a class of visionary men, who waste their lives in a vain attempt at the realization of Utopian dreams.

The transactions also contain the report of the committee on Medical Literature—a paper on Diatheses, their Surgical relations and effects, by Prof. E. Andrews—one on the American Method of Treating Joint Diseases and Deformities, by Henry G. Davis, M. D.—the Report of a case of Diarrhœa Adiposa, by John H. Griscom, M. D.—Report on American Medical Necrology, by Dr. Christopher C. Cox—A paper on Laryngoscopal Therapy, or the Medication of the Larynx under Sight, by Louis Elsberg, M. D.—An Essay on the Veratrum Viride, for which the Association awarded the annual prize, is by Prof. Samuel R. Percy. It is very ample, and we have neither time nor space to notice it at present.

---

*The People's Dental Journal.*—Edited by Drs. W. W. Allport, A. Hill, and J. Richardson. Quarterly. Price $1,00 a year. Chicago.

The initial number of the second volume of this candidate for popular favor comes to us improved in appearance. It contains much valuable information for the people in regard to "keeping the mouth in a healthy and working condition." It is commended by the leading Dental Journals; and we therefore recommend it to the attention of those having an interest in the subject, and who has not?

---

*The Alleged Case of Poisoning by a Medical Man in Paris.*—We mentioned a short time since that a homœopath, of Paris, had been taken into custody upon the charge of poisoning a lady, whose life was insured in his favor for £22,000. The case, according to French law, is being investigated by the Judge specially entrusted with the preliminary steps in the trial (juge d'instruction), the prisoner being all the while under close arrest. It would appear that these investigations have led to disclosures which throw additional suspicions upon the prisoner respecting the death of his mother-in-law, which took place two years ago. A great many witnesses have been examined, and careful analysis made by medical men, upon the directions of the judge. The investigation is not yet concluded. The prisoner does not seem much affected by his incarceration and the impending trial. Nay, his activity, vivacity, and petulence seem on the increase, as he is very busy with a lengthy correspondence and satirical writings, in which the persons principally engaged in the investigation are very roughly handled.

